# HTLV screening of blood donors using chemiluminescence immunoassay in three major provincial blood centers of China

**DOI:** 10.1186/s12879-020-05282-2

**Published:** 2020-08-06

**Authors:** Junpeng Zhao, Feixue Zhao, Wei Han, Xiaoxuan Xu, Lilin Wang, Ran Li, Tong Li, Linfeng Wu, Dandan Du, Xuezhen Zeng, Xiujuan Cui, Yunlong Chen, Jinfeng Zeng, Lunan Wang

**Affiliations:** 1 Shenzhen blood center, Meigang South Road, Shenzhen, People’s Republic of China 518000; 2 Changsha blood center, Changsha, People’s Republic of China; 3 Hebei blood center, Shijiazhuang, People’s Republic of China; 4 National Center for Clinical Laboratories, Beijing Hospital, National Center of Gerontology; Institute of Geriatric Medicine, Chinese Academy of Medical Sciences, Beijing, People’s Republic of China

**Keywords:** Human T-cell lymphotropic virus (HTLV), Blood donors, Chemiluminescence immunoassay (CLIA), Blood screening, Prevalence

## Abstract

**Background:**

Human T-cell lymphotropic virus (HTLV) remains a major safety concern for blood supplies. Despite many HTLV positive cases being reported in southeastern China, the detection of HTLV has not been prioritized in routine blood screening. Additionally, data on the prevalence of HTLV infection among blood donors is also limited. The objective of this study was to investigate the prevalence of HTLV among blood donors in three Chinese provinces through their representative blood centers, to evaluate the feasibility of chemiluminescence immunoassay (CLIA) for blood screening.

**Methods:**

From November 2018 to March 2019, blood plasma samples were collected from Hebei, Changsha, and Shenzhen blood centers and were screened for the HTLV-1/2 antibody using a CLIA and enzyme-linked immunosorbent assay (ELISA). This was followed by confirmatory tests using INNO-LIA HTLV I/II.

**Results:**

A total of 59,929 blood donations were collected and screened for HTLV-1/2. The reactive rate of CLIA and ELISA among donations in the Shenzhen blood center (0.0943%, 27/28,621) was higher than Hebei (0.0248%, 4/16,144), and Changsha (0.0198%, 3/15,164) (*p* < 0.05). After confirmation, 3 samples were confirmed as indeterminate for HTLV antibodies, and only one sample from the Shenzhen blood center was confirmed as HTLV-1. The overall prevalence of HTLV-1/2 was 1.67 per 100,000 (1/59,929). The HTLV-infected blood came from a 32-year-old first-time female donor with a high school degree, who belonged to the SHE ethnic minority and was born in the Fujian province.

**Conclusions:**

In summary, the overall prevalence of HTLV-1/2 among blood donors in the three blood centers in China remains relatively low. However, blood donations with positive or indeterminate results for HTLV antibodies reminded us of the importance of HTLV screening among blood donors in China.

## Background

The human T-cell lymphotropic virus (HTLV) was the first human retrovirus to be discovered in the early 1980s [1]; It is classified into types 1, 2, 3, and 4 [2]. HTLV-1/2 infection is a high-risk factor for lymphoproliferative and inflammatory conditions and can cause adult T-cell leukemia/lymphoma and HTLV-1-associated myelopathy/tropical spastic paraparesis [3–5]. Currently, both HTLV-3 and HTLV-4 have not been linked to diseases [2]. It is estimated that almost 10–20 million people are infected by HTLV-1/2 globally, according to the report in 2016 [6]; it is transmitted through intravenous drug use, blood transfusion, sexual contact, and from mother to child through breastfeeding [7]. HTLV-1 is mainly endemic to Southwestern Japan, South America, Caribbean islands, Sub-Saharan Africa, Middle East and Austro-Melanesia [8], whereas HTLV-2 is endemic to Africa and South, North, and Central America, mostly found in the Amerindian and Pygmy tribes [9]. The latest data describe that the prevalence of HTLV-1/2 was 2.51 per 100,000 in the major areas of China between January 2016 and December 2017 [10], which is lower than the US, Japan, and European countries [11–14].

Several developed countries including the US, France, Netherlands, Sweden, Switzerland, and Japan have conducted HTLV screening among blood donors with different screening strategies based on its prevalence. HTLV-1/2 infection has not been included as part of routine blood screening at the national level. In accordance with the requirements of the National Health Commission in China, laboratories in the Fujian, Zhejiang, and Guangdong provinces, which have a high prevalence of HTLV, do HTLV-1/2 antibody screening on all blood donations. In contrast, other provinces or municipalities have been screening only 10% of the donations for HTLV, since 2015. The prevalence of HTLV in Fujian, Guangdong, and Zhejiang provinces was 24.3 per 100,000 [15], 29.0 per 100,000 [16], 9.1 per 100,000 [17], respectively. Unfortunately, HTLV can potentially spread throughout China from the southeastern region (which has a high prevalence of HTLV) due to the migration of population [10]. Both men and women aged from 18 to 55 years old are allowed to donate blood, a stable pool of regular blood donor account for 30% of all blood donors. Blood donors will accept health inquiry before donating blood, most of them are healthy people and representative for the general population [18, 19].

In China, the data available for HTLV screening among blood donors are limited. Blood screening laboratories conduct HTLV screening using enzyme-linked immunosorbent assay (ELISA), according to the requirement of the Blood Donation Law. The chemiluminescence immunoassay (CLIA), sample pre-processing system, and result analysis system are fully automated and self-contained platforms that minimize operator involvement, have good reproducibility, and can avoid the false positive/negative factors brought by operators [20, 21]. This study investigated the prevalence of HTLV-1/2 among blood donors in three blood centers (South region: Shenzhen blood center; Central region: Changsha blood center; North region: Hebei blood center), using CLIA and ELISA at the same time.

## Methods

**Fig. 1:**
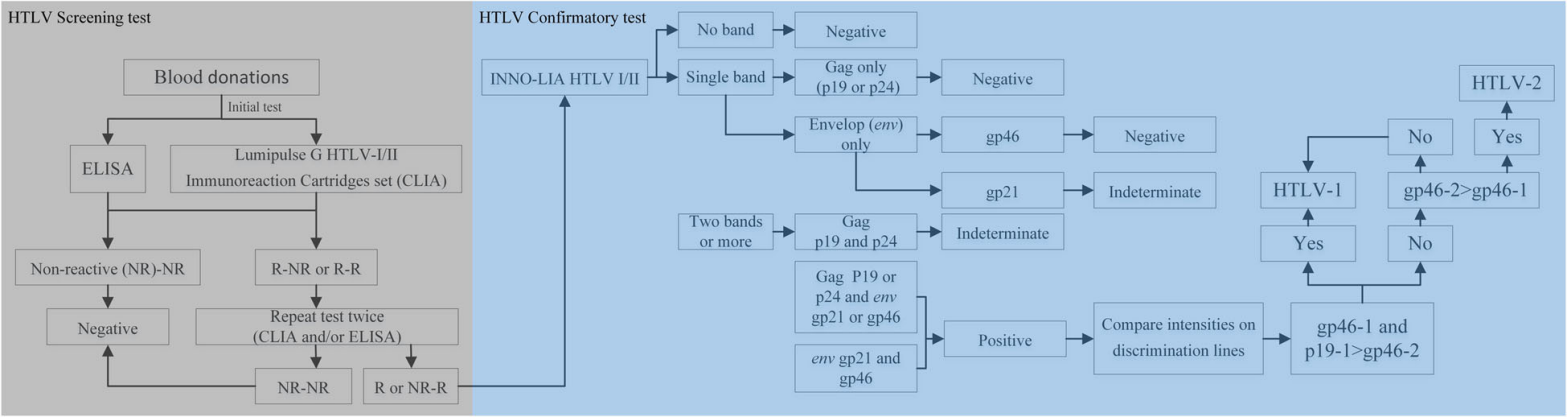
The study routes of HTLV screening test and confirmatory test

From November 2018 to March 2019, plasma samples from Changsha and Hebei blood centers underwent ELISA using the diagnostic kit for HTLV-1/2 antibodies (Beijing Wantai Biological Pharmacy, Beijing, China) and blood plasma samples in Shenzhen were screened by Murex HTLV I + II (DiaSorin, Saluggia, Italy). After ELISA screening, blood donations were randomly selected and screened for the HTLV-1/2 antibody using LUMIPULSE G HTLV-I/II Immunoreaction Cartridges and LUMIPULSE L2400 (Fujirebio, Europe, N. V, Belgium) in their respective blood centers, according to the number of tests. The number of blood donations was equal to the number of blood donors. The sample with initial ELISA and/or CLIA reactivity will perform repeated tests twice using the same reagent. If one of the two outputs was identified as HTLV-1/2 reactive, it was followed up with a confirmatory test using INNO-LIA HTLV I/II (Fujirebio, Europe, N. V, Belgium); the final results were analyzed by Auto-LIA 48 automatic system (Fujirebio, Europe, N. V, Belgium). The study route and criteria for interpreting the results obtained with the INNO-LIA strips were described in Fig. 1. All the indeterminate cases were followed up with a confirmatory test using INNO-LIA HTLV I/II at a 3-month interval.

Since laboratories in China have not been performing routine HTLV screening, the donors that were found to be initially reactive for HTLV antibodies using ELISA were not deferred until they were confirmed to be positive for HTLV in National Centers for Clinical Laboratories using western blot or INNO-LIA HTLV I/II, according to the screening algorithm. However, the donation that was detected as reactive in ELISA was discarded.

According to the requirements of the National Health Commission in China, all blood donations underwent HTLV screening using ELISA in the Shenzhen blood center, while blood centers of Changsha and Hebei blood screened only 10% of the blood donations. However, during the course of this study, HTLV screening was conducted for all blood donations across all three laboratories. Furthermore, these samples were also screened for Hepatitis B surface antigen, antibody to Hepatitis C Virus, antibody to treponema pallidum, and antibody to human immunodeficiency virus (HIV) and/or HIV-1 P24 antigen using ELISA; donations with reactive results for the serologic markers were not excluded from this study.

### Statistical analyses

The SPSS 21.0 software was used for statistical analysis. Chi-square tests were performed on all the examined outcomes between the three blood screening laboratories; *p <* 0.05 was considered statistically significant.

## Results

### HTLV screening test

**Fig. 2:**
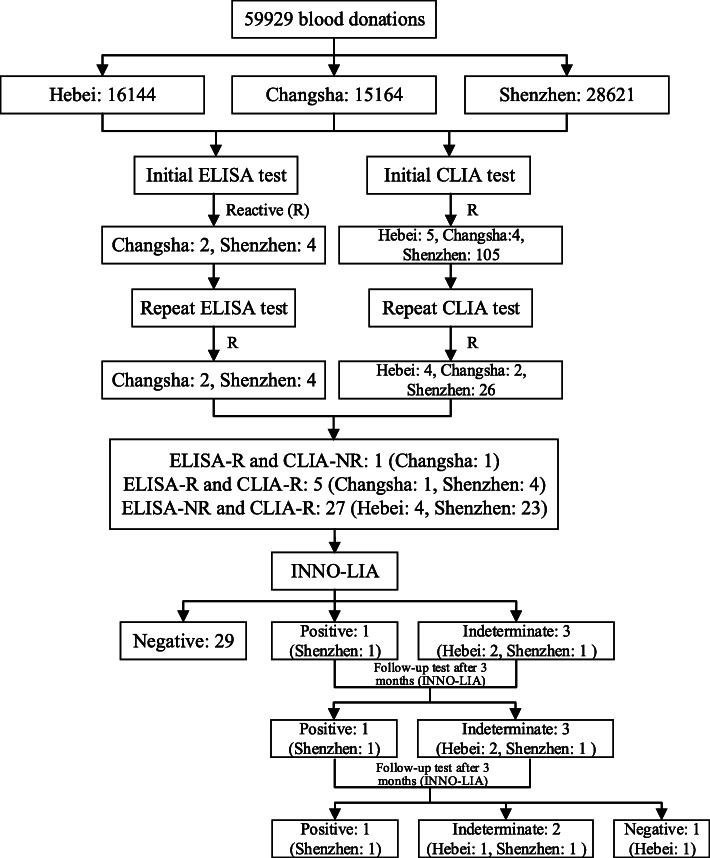
The details of HTLV screening strategy in the study

A total of 59,929 blood donations (Hebei: 16,144, Changsha: 15,164, Shenzhen: 28,621) were collected and screened for HTLV-1/2 using CLIA (Table 1). Among these, 115 were initially detected as reactive in ELISA and/or CLIA, of which one sample was only reactive in ELISA but non-reactive in CLIA; 109 donations were reactive in CLIA and non-reactive in ELISA, and 5 samples were detected as reactive in ELISA and CLIA (Fig. 2). After repeated testing, 33 blood donations (Hebei: 4, Changsha: 3, Shenzhen: 26) were identified as reactive for the HTLV antibody (Table 1). The repeated reactive results in CLIA and/or ELISA among donations in the Shenzhen blood center (0.0908%, 26/28,621) was higher than Hebei (0.0248%, 4/16,144) and Changsha (0.0198%, 3/15,164) (*p* < 0.05); no significant difference in serologic activity was found between Hebei and Changsha blood centers.

**Table 1:** The data of HTLV-1/2 screening using CLIA and ELISA among blood donors in the three blood screening laboratories

S/CO of CLIA	Hebei blood center		Changsha blood center		Shenzhen blood center		Sum	
	Initial test	Retest	Initial test	Retest	Initial test	Retest	Initial test	Retest
0.1	2529	/	2675	/	2930	/	8134	/
0.2	6996	/	6433	/	12,707	/	26,136	/
0.3	4341	/	4335	/	8712	/	17,388	/
0.4	1582	/	1276	/	2997	/	5855	/
0.5	490	/	318	/	861	/	1669	/
0.6	142	/	88	/	214	/	444	/
0.7	46	/	27	/	65	/	138	/
0.8	10	/	5	/	22	/	37	/
0.9	3	/	3	/	8	/	14	/
≥1.0	5	4	4	2	105	26	114	32
Number of donations	16,144		15,164		28,621		59,929	
L2400 non-reactive	16,140 (99.9752%)		15,162 (99.9868%)		28,595 (99.9092%)		59,897 (99.9466%)	
L2400 reactive	4 (0.0248%)		2 (0.0132%)		26 (0.0908%)		32 (0.0534%)	
ELISA non-reactive	16,144 (100.0000%)		15,162 (99.9868%)		28,617 (99.9860%)		59,923 (99.9900%)	
ELISA reactive	0 (0.0000%)		2 (0.0132%)		4 (0.0140%)		6 (0.0100%)	
INNO-LIA negative	2		3		24		28	
INNO-LIA positive	0		0		1		1	
INNO-LIA indeterminate	2		0		1		3	
Indeterminate rate	0.0124%		0.0000%		0.0035%		0.0050%	
Negative rate	99.9876%		100.0000%		99.9930%		99.9933%	
Positive rate	0.0000%		0.0000%		0.0035%		0.0017%

### HTLV confirmatory test

Among the 33 reactive blood donations for HTLV-1/2 by L2400 and/or ELISA, 29 were confirmed as HTLV-1/2 negative, 3 were HTLV-1/2 indeterminate (Hebei: 2, Shenzhen: 1), and only one collected in Shenzhen was positive for HTLV-1 antibodies (Table 2). Among the 6 samples that were initially reactive in ELISA, one (16.7%, 1/6) was confirmed as HTLV positive and one (16.7%, 1/6) was indeterminate. Out of 32 samples were detected reactive in CLIA, one was positive (3.1%, 1/32) and 2 samples (6.3%, 2/32) with indeterminate results were observed after the INNO-LIA test (Fig. 2). Indeterminate HTLV-1/2 samples in Hebei, Changsha, and Shenzhen blood centers consisted of 0.0124% (2/16,144), 0.0000% (0/15,164), and 0.0035% (1/28621) of the samples, respectively (*p* > 0.05). The true negative population percentage of HTLV-1/2 among blood donors in Hebei was 99.9752% (16,140/16,144), which was similar to Changsha (99.9868%, 15,162/15,164) and Shenzhen (99.9092%, 28,595/28,621) (*p* > 0.05). Furthermore, no statistical differences were observed in the HTLV-infected donors in the three blood screening laboratories (*p* > 0.05). In summary, of the 59,929 donations, 99.9933% (59,925/59,929) samples were confirmed as HTLV-1/2 negative, and 0.0050% (3/59,929) samples were classified as indeterminate for HTLV-1/2 antibodies. The overall prevalence of HTLV-1/2 was 1.67 per 100,000 (1/59,929).

**Table 2:** The details of L2400 and INNO-LIA HTLV I/II Score among blood donations with reactive results for HTLV-1/2

Code	ELISA	L2400				INNO-LIA HTLV I/II Score							
	S/CO mean (Retest)	S/CO (Initial test)	S/CO (Retest)		Result	Confirmation				Discrimination			Result
			*n* = 1	*n* = 2		p19 I/II	p24 I/II	gp46 I/II	gp21 I/II	p19I	gp46I	gp46II	
Hebei-1	–	7.5	7.3	7.4	reactive	–	–	2+	–	–	–	2+	negative
Hebei-2	–	1.8	1.6	1.6	reactive	–	–	–	1+	–	–	–	**indeterminate**
Hebei-3	–	6.1	6.0	6.2	reactive	–	–	–	–	–	–	–	negative
Hebei-4	–	2.4	2.5	2.6	reactive	–	–	–	2+	–	–	–	**indeterminate**
Changsha-1	1.8	–	–	–	reactive	–	–	–	–	–	–	–	negative
Changsha-2	2.3	1.1	1.2	1.2	reactive	–	–	–	–	–	–	–	negative
Changsha-3	–	2.3	1.2	1.2	reactive	–	–	–	–	–	–	–	negative
Shenzhen-1	–	1.5	1.3	1.3	reactive	–	–	–	–	–	–	–	negative
Shenzhen-2	–	3.8	3.4	3.4	reactive	–	–	–	–	–	–	–	negative
Shenzhen-3	–	3.1	3.1	3.0	reactive	–	–	–	–	–	–	–	negative
Shenzhen-4	–	1.3	1.4	1.4	reactive	–	–	–	–	–	–	–	negative
Shenzhen-5	–	50.0	50.0	50.0	reactive	–	–	1+	–	–	1+	–	negative
Shenzhen-6	–	50.0	50.0	50.0	reactive	–	–	–	–	–	–	–	negative
Shenzhen-7	–	1.3	1.3	1.3	reactive	–	–	–	–	–	–	–	negative
Shenzhen-8	–	1.6	1.6	1.6	reactive	–	–	–	–	–	–	–	negative
Shenzhen-9	–	1.1	1.0	1.0	reactive	–	–	–	–	–	–	–	negative
Shenzhen-10	–	1.2	1.2	1.2	reactive	–	–	–	–	–	–	–	negative
Shenzhen-11	–	1.9	1.9	2.0	reactive	–	–	–	–	–	–	–	negative
Shenzhen-12	11.63	50.0	50.0	50.0	reactive	2+	2+	3+	2+	2+	2+	–	**positive**
Shenzhen-13	–	1.0	0.9	1.0	reactive	–	–	–	–	–	–	–	negative
Shenzhen-14	–	2.6	2.6	2.5	reactive	–	–	–	–	–	–	–	negative
Shenzhen-15	–	1.7	1.8	1.8	reactive	–	–	–	–	–	–	–	negative
Shenzhen-16	–	2.3	2.3	2.4	reactive	–	–	–	–	–	–	–	negative
Shenzhen-17	–	1.2	1.0	1.1	reactive	–	–	–	–	–	–	–	negative
Shenzhen-18	–	2.1	2.0	2.0	reactive	–	–	–	–	–	–	–	negative
Shenzhen-19	6.89	3.5	3.6	3.7	reactive	–	–	–	1 + −	–	–	–	**indeterminate**
Shenzhen-20	–	1.4	1.3	1.3	reactive	–	–	–	–	–	–	–	negative
Shenzhen-21	–	1.1	1.0	1.0	reactive	–	–	–	–	–	–	–	negative
Shenzhen-22	2.27	1.0	1.1	1.0	reactive	–	–	–	–	–	–	–	negative
Shenzhen-23	–	1.4	1.3	1.3	reactive	–	–	–	–	–	–	–	negative
Shenzhen-24	–	1.3	1.1	1.1	reactive	–	–	1+	–	–	1+	–	negative
Shenzhen-25	–	1.7	1.6	1.6	reactive	–	–	–	–	–	–	–	negative
Shenzhen-26	3.7	3.0	2.9	3.1	reactive	–	–	–	–	–	–	–	negative

No changes were found in the results of the indeterminate and positive cases after the follow-up test in three months, while one indeterminate case from Hebei tested negative in the second follow-up test (Fig. 2).

The demographic characteristics showed that the blood donor with HTLV-1 infection in the study was a 32-year-old first-time female donor from the Fujian province who belonged to the SHE ethnicity minority and had a high school degree. She was unmarried and had three sexual partners with unprotected sex. In addition, she once pierced her ear, received acupuncture therapy, and had endoscopy twice. Three blood donors with indeterminate results belonged to the HAN ethnicity. Most of the indeterminate cases were unmarried workers and first-time donors with associate degrees (Table 3).

**Table 3:** Demographic characteristics of blood donors with reactive or indeterminate results by INNO-LIA HTLV I/II Score

Code	Result of INNO-LIA	Blood center	Native place	Gender	Marital status	Age range	Previous donation history	Ethnicity	Education	Occupation	Follow-up test of INNO-LIA	
											First time (3 months)	Second time (3 months)
Shenzhen-12	Positive	Shenzhen	Fujian Ningde	Female	Unmarried	26–35	First-time	SHE	High school degree	Worker	Positive	Positive
Shenzhen-19	Indeterminate	Shenzhen	Guangdong Meizhou	Male	Unmarried	18–25	First-time	HAN	Associate degree	Worker	Indeterminate	Indeterminate
Hebei-2	Indeterminate	Hebei	Hebei Handan	Female	Unmarried	18–25	First-time	HAN	Associate degree	Student	Indeterminate	Negative
Hebei-4	Indeterminate	Hebei	Hebei Shijiazhuang	Male	Married	46–55	Repeated	HAN	High school degree	Worker	Indeterminate	Indeterminate

The L2400 and INNO-LIA HTLV I/II score among blood donations varied. Out of 32 plasma samples reactive on CLIA, 27 (84.4%) had sample cutoff (S/CO) values between 1.0 and 5.0, and 5 (15.6%) had S/CO values > 5.0. Regarding the confirmatory INNO-LIA tests, seven samples had lines upon confirmation (*gag* p19 I/II, *gag* p24 I/II, *env* gp46 I/II, *env* gp21 I/II). Of these, 3 donations with a single gp46 I/II band were identified as negative for HTLV antibodies, while 3 samples with single gp21 I/II were classified as indeterminate for antibodies. Only one donation had the 4 confirmation lines and was confirmed as HTLV-1 positive due to the 2 bands of discrimination (p19 I and gp46 I).

## Discussion

Prior to our study, laboratories were not performing HTLV-1/2 screening on route blood donations according to the Blood Donation Law in China. This study conducted HTLV-1/2 screening among blood donors and reported the prevalence of HTLV-1/2 among blood donors in three blood centers located in the southern (Shenzhen blood center), central (Changsha blood center), and northern regions (Hebei blood center) of China.

From November 2018 to March 2019, no HTLV-infected blood donors were found in Changsha and Hebei, and only one blood donor from Shenzhen was positive for HTLV-1 antibodies. Both the CLIA and the ELISA tests detected the one seropositive sample in this study. In addition, there were not many false-positive results on CLIA testing and even less on ELISA testing. In other words, both tests had a high specificity. The prevalence of HTLV-1 among blood donors in Shenzhen was 0.0035%, which was similar to the latest survey on HTLV prevalence (0.0029%) among volunteer blood donors in Shenzhen, conducted by Institute of Blood Transfusion attached with Chinese Academy of Medical Sciences [10], which found six infected donor from the 208,647 donors tested in Shenzhen from January 2016 to December 2017. Moreover, the sample size of blood donors screened by LUMIPULSE G HTLV-I/II Immunoreaction Cartridges was quite small to lend insight into such an epidemic. In the present study, the nonspecific reactive rate of CLIA or ELISA in the blood centers of Changsha and Hebei was lower than Shenzhen, which might be associated with the prevalence of HTLV; a similar phenomenon was found in an epidemiological study of HIV in an area of low prevalence [22]. Furthermore, required by the donor deferral guideline in China, blood donors that test reactive for anti-HTLV-1/2 in ELISA on only one occasion are not deferred unless further confirmatory testing is positive, which may impact the study data. According to the socio-demographic information, we found that the only HTLV-1-infected donor in Shenzhen came from the Fujian province; a 32-year-old first-time female donor of the SHE ethnic minority, with a low educational background. This was the first time an HTLV-infected blood donor of the SHE minority was reported in China.

The Fujian province has highest prevalence of HTLV in China [17]. However, Shenzhen as a city has the largest population flow; it is located in the Guangdong province, which has the second highest HTLV prevalence in China [17]. Hence, Shenzhen may have more blood donors with HTLV infections, as compared to Changsha and Hebei. Furthermore, the HTLV-infected donor in this study had several sex partners and underwent acupuncture theory and endoscopy; unprotected sex and traumatic operations may increase the risk of HTLV transmission [23, 24]. After two follow-up tests, one indeterminate case from Hebei changed into negative, while no changes were found in the results of the 2 indeterminate cases and one positive case, which may be related to the low antibody titer of the donor and the sensitivity and specificity of INNO-LIA [25]. Although one sample was confirmed as HTLV-1 positive, 6 donations had the confirmation lines in the INNO-LIA HTLV I/II score. Of these, 3 samples were indeterminate due to a high possibility of HTLV antibodies. An indeterminate result for the INNO-LIA HTLV I/II score needs a follow-up test, and additional testing such as polymerase chain reaction is recommended by the manufacturer. Among the 32 samples that were found to be reactive in CLIA, the majority of samples had an S/CO value of 1.0–5.0, 3 samples were strongly reactive for HTLV-1/2 in CLIA (S/CO ≥ 50), but 2 samples were detected non-reactive in ELISA, and identified as negative for HTLV-1/2 antibodies after the INNO-LIA test. The confirmed HTLV-1-positive sample (ID: Shenzhen-12) was the one of these three samples with high S/CO levels. Mutations in testing targets may have resulted in the missing of INNO-LIA scores. Furthermore, one sample with an indeterminate result by INNO-LIA tested reactive in CLIA but was missed in ELISA, even though ELISA had a higher specificity than CLIA in this study, which reflected that CLIA might have higher sensitivity than ELISA [26].

### Limitations

Our study did have a few limitations. Firstly, although highly sensitive CLIA can be used for routine blood screening [26], HTLV screening among blood donors with CLIA cannot last for a long time due to the high costs, unless it can get the support of the government and manufacturer. The reagent of CLIA (L2400) has not been approved by China Food and Drug Administration, it costs about 10 dollars per test in Japan, which is higher than the cost of ELISA (about 1 dollar per test), but its cost can be cut down through large package. Secondly, although the sample size was large, the prevalence of HTLV was small in this population; hence, the precision of the epidemiological estimate was low. Thirdly, all the blood donations with non-reactive results in CLIA and ELISA were not further confirmed by INNO-LIA, which may lead to the undetected HTLV infection. Lastly, the blood screening laboratories that we considered for this study were also limited.

## Conclusion

In summary, the overall prevalence of HTLV-1/2 among blood donors in the three major provincial blood centers in China was 0.0017%, which is lower than Japan, the US, and many European countries that have implemented HTLV donor testing. However, blood donations that had positive or indeterminate results for HTLV antibodies reminded us of the importance of HTLV screening among blood donors in China to prevent the transmission of HTLV from blood transfusion. In the view of HTLV prevalence and cost concerns, we support the view that HTLV screening of blood donors in area with high prevalence is reasonable in China; all the first-blood donors should undergo HTLV screening as a systematic strategy in low prevalence of HTLV. We recommend that the future research based on this study must focus on an expanding sample size and geographical coverage to get a more comprehensive dataset on HTLV prevalence, to ultimately improve blood safety.

## Data Availability

The data for this study is available from the corresponding author on reasonable request.

## Abbreviations

HTLV: Human T-cell lymphotropic virus
CLIA: Chemiluminescence immunoassay
ELISA: Enzyme-linked immunosorbent assay
HIV: Human immunodeficiency virus
S/CO: Sample cutoff
